# Evolution of Human Adenoviruses, a Double-Stranded DNA Viral Pathogen Documented Through Genomics and Bioinformatics and Viewed Through a Web Resource Database

**DOI:** 10.3390/v18020251

**Published:** 2026-02-16

**Authors:** Katayoon Dadkhah, Shoaleh Dehghan, James Chodosh, Qiwei Zhang, Donald Seto

**Affiliations:** 1 Bioinformatics and Computational Biology Program, School of Systems Biology, George Mason University, Manassas, VA 20110, USA; kdadkhah@gmu.edu; 2 Chemistry Department, American University, Washington, DC 20016, USA; shoalehd@gmail.com; 3 Department of Ophthalmology & Visual Sciences, University of New Mexico, Albuquerque, NM 87106, USA; jchodosh@salud.unm.edu; 4 Key Laboratory of Viral Pathogenesis & Infection Prevention and Control, Ministry of Education, Institute of Medical Microbiology, College of Life Science and Technology, Jinan University, Guangzhou 510632, China; zhangqw@jnu.edu.cn

**Keywords:** human adenovirus, viral pathogen, genomics, classification, viral taxonomy, database, web resource, virus evolution, molecular evolution, zoonosis, double-stranded DNA viruses, White King Reigns hypothesis

## Abstract

Human adenoviruses (HAdVs) remain prominent global human pathogens, particularly in dense, crowded populations. The advent of genomic and bioinformatic tools allows for high-resolution means to identify, characterize, and understand these pathogens. These tools also provide the basis for the standardization of names, as well as an accessible archive of all genotypes (“Human Adenovirus Working Group”). This overview and perspective of all the genotypes in one setting provides a better understanding of the mechanisms of their molecular evolution: genome recombination plays a major role in the emergence of novel adenoviral pathogens. In the context of the fidelity of their DNA polymerase replication machinery, this strategy provides entry into immune-naïve host populations through the acquisition of genome sequences that may include antigenic epitopes that have not circulated commonly, widely, or recently, as well as sequences encoding host cell entry proteins. Using the “chess” metaphor for describing the rapid evolution of RNA viruses, we propose a similar but diametrically opposed “White King Reigns in the Family of Human Adenoviruses”.

## 1. Introduction

Adenoviruses (AdVs) are non-enveloped, double-stranded DNA viruses that infect a wide spectrum of vertebrate species [1]. Human adenoviruses (HAdVs) were first isolated and characterized in the early 1950s as respiratory pathogens [2,3] and subsequently identified as significant ocular, gastrointestinal, and renal pathogens that are still prominent and potentially troublesome pathogens with respect to global public health [4]. It was categorized recently as a “Low–Medium” Public Health Emergencies of International Concern (PHEICs) risk in a June 2024 publication by the World Health Organization (WHO)’s Research & Development Blueprint for Epidemics group entitled “Pathogens Prioritization: A Scientific Framework for Epidemic and Pandemic Research Preparedness” (www.who.int, accessed on 1 February 2026) [5]. Examples of recent, large, and extended outbreaks with significant morbidity and mortality impacts include HAdV-B55 at various locations in China [6,7]; HAdV-B3, HAdV-B3/B7, and HAdV-B7/B3 in Kolkata and surrounding districts (India) [8,9]; and HAdV-B3 and -B7 in the USA [10]. Additionally, with genomic sequencing and characterization, HAdVs are significant pathogens for individuals, e.g., immunocompromised individuals [4,11,12].

A critical step in managing and limiting outbreaks, particularly pandemics, is the identification and characterization of the pathogen, determining specifically whether it is an unknown, novel, and emergent pathogen or a known but previously non-circulating and perhaps recently evolved, re-emergent microbe [13]. As demonstrated in the recent pandemic with SARS-2, the genome sequence [14] is absolutely critical for initiating both diagnostic assay [15] and vaccine development [16,17], as well as for implementing suitable public health measures to limit the consequences [18]. With the discussions of using the high-resolution genome data as opposed to serology-based methods as the basis for identifying, characterizing, and naming these long-studied pathogens largely settled [19,20,21], the establishment of appropriate public resources for cataloging and archiving recent as well as previously accepted HAdV prototypes was important. Evaluating claims for novel genotype candidates in an orderly fashion was also needed, for example, to avoid simultaneous reports of novel genotypes, e.g., HAdV-D53 [22], and to avoid potentially “novel” but erroneous genotype recognition [9] and to prevent GenBank deposits of laboratory-generated vectors and adenoviruses (D.S., *pers. observation*). In collaboration with J. Rodney Brister of the National Center for Biotechnology Information in the National Library of Medicine, at the National Institutes of Health, a working group of human adenovirus researchers (James Chodosh, Albert Heim, Morris S. Jones, Adriana E. Kajon, Thomas Lion, Donald Seto, and Qiwei Zhang) was established in 2011 to vet candidates as novel genotypes for archiving into GenBank and for assigning and recording new genotype numbers to a public resource site. The e-location for this resource is the “Human Adenovirus Working Group” (http://hadvwg.gmu.edu/, accessed on 1 February 2026), with the most recent genotype recognition of HAdV-116 (March 2024).

One benefit of this resource is a readily available overview of the genomic evolution and emergence of novel HAdV pathogens. Whether these emergent and novel HAdVs evolved through nucleotide divergence or drift, e.g., base substitutions and insertions and deletions (indels), or through larger genome modifications or shift, e.g., recombination, becomes apparent during the evaluation of their whole genome data, including the parsing of the penton base, hexon, and fiber nucleotide sequences as convenient landmarks for the length of the genome. Admittedly this scheme is not ideal as it does not consider additional and important genomic sequences, for example, genome replication optimization [23,24,25], host–pathogen interaction [26,27], and pathogenicity-related regions. However, these three markers have served to characterize novel types of HAdV and to account for differences in pathogenicity and clinical attributes. Additionally, genome analysis provides documentation of novel human HAdV pathogens arising through host species jumps, i.e., zoonosis [28,29,30]. With the overview of genotypes provided by this resource, it is apparent that genome recombination appears to be a significant mechanism in the emergence and re-emergence of human adenoviral pathogens rather than genome drift. In this context, and diametrically opposed to the “it takes all the running you can do to keep in the same place” strategy of the “Red Queen Reigns in the Kingdom of RNA Viruses” exemplified in the 1990s by vesicular stomatitis virus [31], we propose an evolution strategy for this double-stranded adenovirus as the “White King Reigns in the Family of Human Adenoviruses”.

## 2. Materials and Methods

### 2.1. Human Adenovirus Working Group Reference Website

The website is hosted on the “BINF” server housed in the School of Systems Biology at George Mason University (Manassas, VA, USA). This server comprises a Hewlett-Packard xw9400 Workstation (HP Inc., Palo Alto, CA, USA) running Red Hat Enterprise Linux (Red Hat Inc., Raleigh, NC, USA). The processor is an Intel Xeon E5-2630 v2 (2.60GHz), containing a two-core CPU with 8GB of RAM.

The website was built using WordPress (WordPress, 2015), in partial fulfillment of her Ph.D. dissertation, by Dr. Elizabeth B. Liu [32]. It is a basic web content hosting site that provides reference information regarding human adenovirus typing, a table of genome-based genotypes, criteria for a new HAdV genotype and name, instructions regarding how to submit a candidate HAdV, and a serotyping tool. The serotyping tool was constructed using a “DataTables” JavaScript library-based plugin that is available in the WordPress plugin library. This serotyping tool, in theory, displays all potential types corresponding to the query serotype entered by the user. Unfortunately, this tool was determined to be not sufficiently robust and is still under development using the current larger dataset of genes and genomes.

Upon submission of a candidate novel genotype, the request and supporting data files are forwarded automatically to the chair of the Human Adenovirus Working Group, who, in turn, distributes the files to members of the working group. Discussions and voting are held virtually, and a decision is returned to the user. Novel genotypes and characterization data are uploaded onto the site for public access.

### 2.2. Nucleotide Sequence Analysis: Sequence Retrieval and Alignment

Whole genome and penton base, hexon, and fiber gene sequences of 113 human adenoviruses and 16 simian adenoviruses were retrieved from the GenBank database using their published accession numbers. Multiple sequence alignments (MSA) were performed using MAFFT, version 7 (https://mafft.cbrc.jp/alignment/server/, accessed on 28 December 2025), with default parameters. Genome data for HAdV-C89, -D90, and -D116 were not included in deference to their unpublished status in Genbank.

### 2.3. Phylogenetic Analysis

Results from MSA were used for inferring phylogenetic relationships of the whole genomes and the penton base, hexon, and fiber genes of the above-noted adenoviruses using a maximum-likelihood (ML) approach. Phylogenetic analyses were conducted with IQ-TREE 2 [33]. ML tree inference was performed using default settings, except that the best-fit nucleotide substitution model was selected using ModelFinder, and branch support was assessed using ultrafast bootstrap and SH-like approximate likelihood ratio test (SH-aLRT), each with 1000 replicates.

### 2.4. Tree Visualization

Phylogenetic trees were generated using IQ-TREE 2 and are visualized with the Interactive Tree of Life (iTOL) online platform (https://itol.embl.de/, accessed on 28 December 2025) [34]. Trees were uploaded to iTOL for interactive visualization and annotation. Default display settings were used to generate standardized tree representations, illustrating the phylogenetic relationships among human (HAdV) and simian adenovirus (SAdV) sequences for each dataset.

## 3. Results

**Figure 1:**
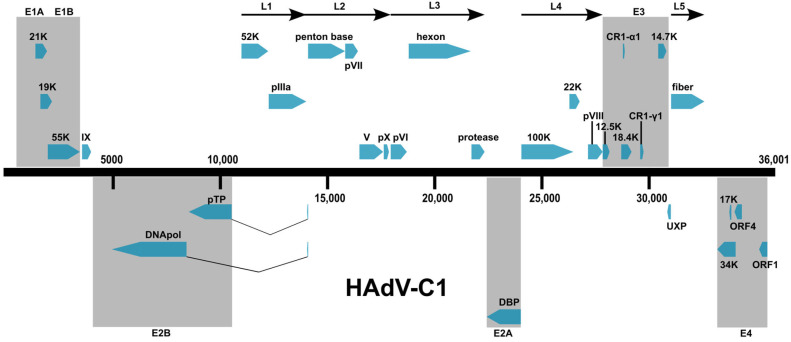
Genome organization of HAdV-C1. The genome is represented by a central black horizontal line marked at 5 kb intervals. Select genes, highlighted in blue and above the black line, are noted and represent proteins that are encoded in the forward direction. Underneath the black line are select genes that are encoded on the reverse orientation. Annotation data are found under the Genbank accession number AF534906.

Human adenoviruses were identified and characterized for many years, in the “pre-genomics” era, using serological methods, first targeting the hexon protein (“epsilon”), the major viral coat protein. This provided for the differentiation of specific serotypes [35,36] based on virus or serum neutralization. A second epitope, located on the fiber protein, the viral coat protein (“gamma”) associated with cell entry and tropism, provided additional characterization and differentiation [35] based on hemagglutination inhibition. This second serological marker provided for the differentiation and rationalization of two epsilon cross-reacting serotypes, e.g., HAdV-E4 and -B16, as their gamma epitopes differed, as well as for HAdV-D15 and -D29 [37]. Prior to the development of the hemagglutination inhibition procedure, the seroneutralization cross-reaction between HAdV-D15 and -D29 was strong enough to provoke discussions as to whether these were separate “types” [35,36,37]. Other examples are found in the early reports; for example, W.P. Rowe, et al., 1958 noted this exception for the two co-circulating “serotyped” HAdV-B7s: “The 7a virus, while closely related to type 7, appears sufficiently distinct immunologically to justify its consideration as a separate adenovirus” [38]. To be consistent with the serological basis of types, gene sequences of the hexon and fiber are used for genome-based typing, as well as sequences of the third viral coat protein, penton base. This may be by whole genome sequencing or by amplifying and sequencing the individual genes [39]. For reference, a gene map for HAdV-C1 is provided (Figure 1), based on annotations from K. P. Lauer, et al. [40]. This genome organization is similar to ones reported from other HAdVs, with variations that may be unique to individual genotypes, e.g., E3 genes.

The original 51 serotypes have been genome-sequenced, sequence-analyzed, and reconfirmed as unique “types”, with the above two pairs noted as genome recombinants that were correctly recognized as separate “*bona fide*” “types”, i.e., different genomes and prototypes, as shown in Table 1. To date, 116 genotypes of HAdVs are archived in Genbank.

### Genomic Sequence Data as “State-of-the Art” for Identification, Typing, and Classification of Recent Novel Isolates of Human Adenoviruses

Prior to sequencing the entire set of previously serotyped genomes, with HAdV-G52 as the first, whole genome sequence determination was instituted for characterizing and identifying putatively novel or emergent, and re-emergent, HAdV isolates and pathogens. Table 2 presents these genotypes HAdV-D53 through -D116 as reference prototypes from this “Genomics Era”. These designations represent a paradigm shift in which whole genome data and phylogenetic analysis form the basis of “typing” [21]. Genome recombinants are recognized as unique “types” and given a formal genotype designation rather than be “lost to time, fading memories, and the avalanche of the literature”. As an example, a respiratory pathogen was isolated in Japan from a dead infant and identified in 1958 as the “Takeuchi” or “AV3-7” or “AV3/7” strain [41]. Subsequent analysis with restriction enzyme digest ladders and serotyping indicate it as an intriguing recombinant with an epsilon epitope (hexon) presenting as both HAdV-B3 and -B7 and a gamma epitope (fiber) as HAdV-B3 [42]. Two isolates of this were identified in a 1995 nation-wide outbreak in Japan [43], and it may circulate currently, for example, misidentified in the extensive 2022–2023 four-month outbreak in Kolkata, India [9].

**Figure 2:**
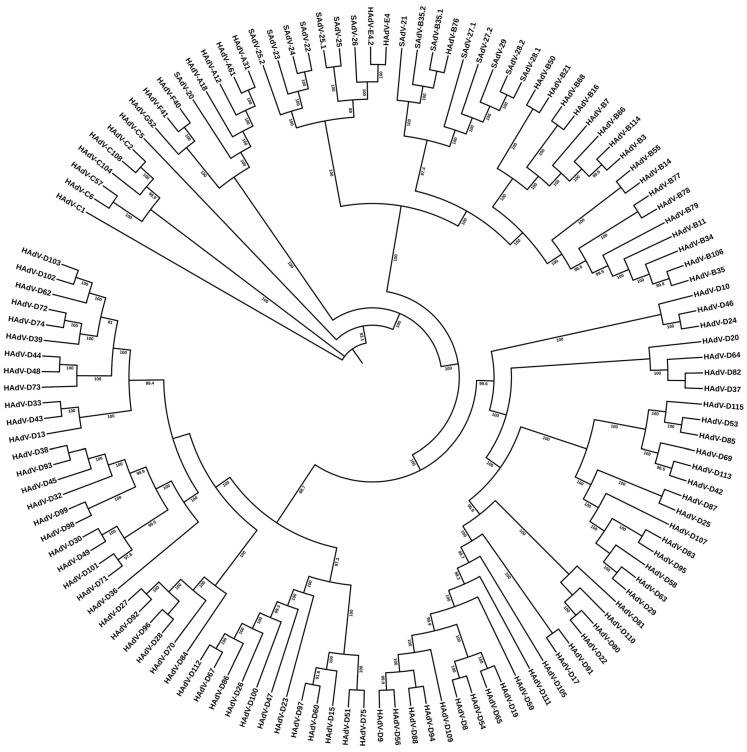
Whole genome sequence-based phylogenetic relationships of human adenovirus genotypes HAdV-C1 through D-115. Genome sequences from 113 human adenoviruses and 16 simian adenoviruses were retrieved from GenBank and aligned using MAFFT version 7, with default settings (https://mafft.cbrc.jp/alignment/server/, accessed on 28 December 2025). Maximum-likelihood (ML) phylogenetic inference was performed using IQ-TREE 2. Only bootstrap support values > 80% are shown. Note: Genome data for HAdV-C89, -D90, and -D116 were not included in deference to their unpublished status in GenBank.

Whole genome sequence data, most accurate when the entire sequence is derived from a single method and by one research group (in contrast to the first GenBank deposits of “mosaic” genomes for HAdV-C2 and -C5 [44,45]), provides high-resolution differentiation of adenovirus prototypes, as noted by the first two HAdV genomes determined in this manner, HAdV-G52 and -C1 [40,46]. With the cascade of additional genome sequencing of all the then-accepted serotypes, select historically important pathogens, and recent emergent pathogens, including resequencing the five HAdVs that were originally deposited in Genbank, one of which, HAdV-D17, being particularly error-riddled [47], bioinformatics analyses yielded detailed sequence similarities and differences, along with phylogenetic relationships [48]. As shown in Figure 2, the whole genome sequences provided demarcation of “types”. Subsequent genome data, including the recent set of genotypes HAdV-D53 through -D116, have reinforced the utility of this high-resolution resource. Simian adenovirus (SAdV) genomes are included for context, particularly for HAdV-E4, the only human adenovirus included in the clade “species E” and reported as a zoonotic pathogen [25]. It is emphasized that a taxonomy-based discussion of these genome-derived data, excluding HAdV-D113, -B114, and -D116, which were identified after 2020, has been presented by J. Kang, et al., 2020 [49], as well as in the individual reports describing each novel genotype from members of the human adenovirus research community, for example, HAdV-C104 [50].

**Figure 3:**
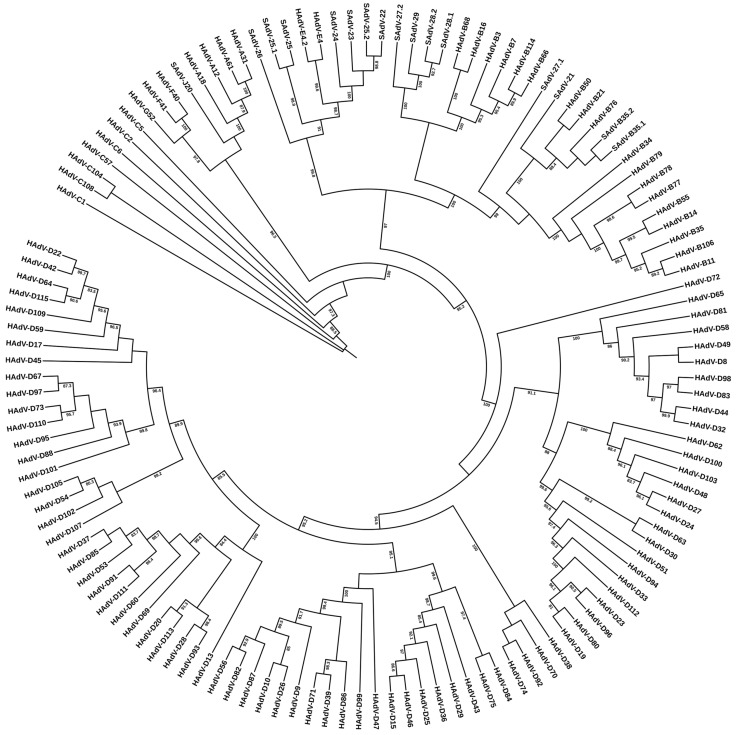
Penton base gene sequence-based phylogenetic relationships of human adenovirus genotypes HAdV-C1 though D-115. Gene sequences from 113 human adenoviruses and 16 simian adenoviruses were retrieved from GenBank and aligned using MAFFT version 7, with default settings (https://mafft.cbrc.jp/alignment/server/, accessed on 28 December 2025). Maximum-likelihood (ML) phylogenetic inference was performed using IQ-TREE 2. Only bootstrap support values > 80% are shown. Note: Genome data for HAdV-C89, -D90, and -D116 were not included in deference to their unpublished status in GenBank.

To be consistent with earlier serological targets for HAdV typing, the third major capsid protein, penton base, was included in the scheme for typing by genome data. Likely, it had not been considered as a marker for HAdV typing as immunochemical methods for the penton base were not available/developed. The L3 penton base gene is located at the 5′ region, approximately 13,000 to 16,000 nucleotides into the genome, which has been noted as ranging from 34 to more than 37 Kb in size [4]. Specifically, for HAdV-C1, the penton base gene is located 14,166 nucleotides into its 36,001 base genome [40]. In addition to serving as a third marker for noting sequence diversity, the penton base is useful as a landmark for genome recombination. As shown in Figure 3, both original serotypes and more recent genotypes contain penton base sequences that complement the whole genome data relationships. HAdV recombinants involving the penton base gene may be identified by comparisons of Figure 3 with the phylogenetic trees parsing the hexon and fiber gene sequences.

**Figure 4:**
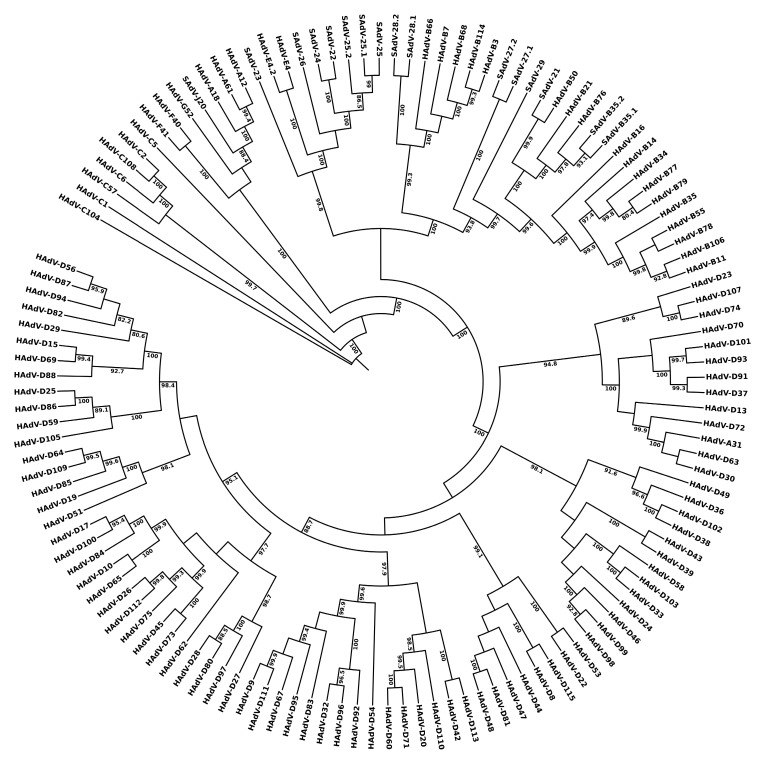
Hexon gene sequence-based phylogenetic relationships of human adenovirus genotypes HAdV-C1 though D-115. Gene sequences from 113 human adenoviruses and 16 simian adenoviruses were retrieved from GenBank and aligned using MAFFT version 7, with default settings (https://mafft.cbrc.jp/alignment/server/, accessed on 28 December 2025). Maximum-likelihood (ML) phylogenetic inference was performed using IQ-TREE 2. Only bootstrap support values > 80% are shown. Note: Genome data for HAdV-C89, -D90, and -D116 were not included in deference to their unpublished status in GenBank.

As noted, the first marker to be used for serologically differentiating HAdVs was the hexon gene, encoding the short sequences that comprise hypervariable loops 1–7 that present as the epsilon epitope [51]. As the predominant component of the virus coat and antigenic, this led to the generation of antibodies from horses and rabbits that provided for the differentiation of specific serotypes [35,36]. These serological differentiations of unique “types” are reconfirmed using the whole hexon sequence data as presented in Figure 4. The L3 hexon gene is located 17,000 to 22,000 nucleotides into the genome, e.g., at 18,861 bases for the 36,001-base HAdV-1 genome [40], providing a landmark for genome recombination events. In the context of adenovirus genomes, it is noted that the hexon sequence, let alone the epitopic sequences, is a very small and misleading contribution to the identity and biology/pathogenicity of athevirus, as noted in a 2012 report titled “Overreliance on the hexon gene leading to misclassification of human adenoviruses” [52]. HAdV recombinants involving the hexon gene may be recognized by comparisons of Figure 4 with the phylogenetic trees parsing the penton base and fiber gene sequences. Again, this is supported by more rigorous analysis [49].

**Figure 5:**
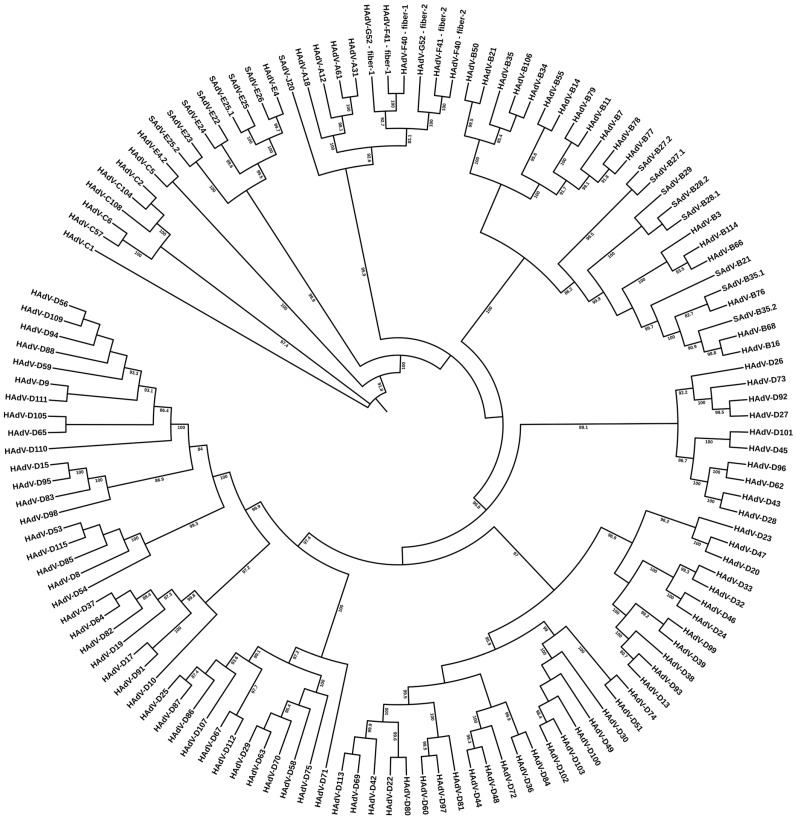
Fiber gene sequence-based phylogenetic relationships of human adenovirus genotypes HAdV-C1 though D-115. Gene sequences from 113 human adenoviruses and 16 simian adenoviruses were retrieved from GenBank and aligned using MAFFT version 7, with default settings (https://mafft.cbrc.jp/alignment/server/, accessed on 28 December 2025). Maximum-likelihood (ML) phylogenetic inference was performed using IQ-TREE 2. Only bootstrap support values > 80% are shown. Note: Genome data for HAdV-C89, -D90, and -D116 were not included in deference to their unpublished status in GenBank.

The fiber gene, providing the gamma epitope for serological typing [35,53], is located towards the 3′ end of the genome, at approximately 29,000 to 32,000 nucleotides or 31,101 for HAdV-1, with a genome size of 36,001 bases [40]. This also serves as a convenient recombination marker at the 3′ end of the genome. As displayed in Figure 5, fiber genes align well due to sequence similarities but parse differentially due to variable and divergent sequences. Recombinants involving the fiber gene may be recognized by comparisons of Figure 5 with the phylogenetic trees parsing the penton base and hexon gene sequences.

**Figure 6:**
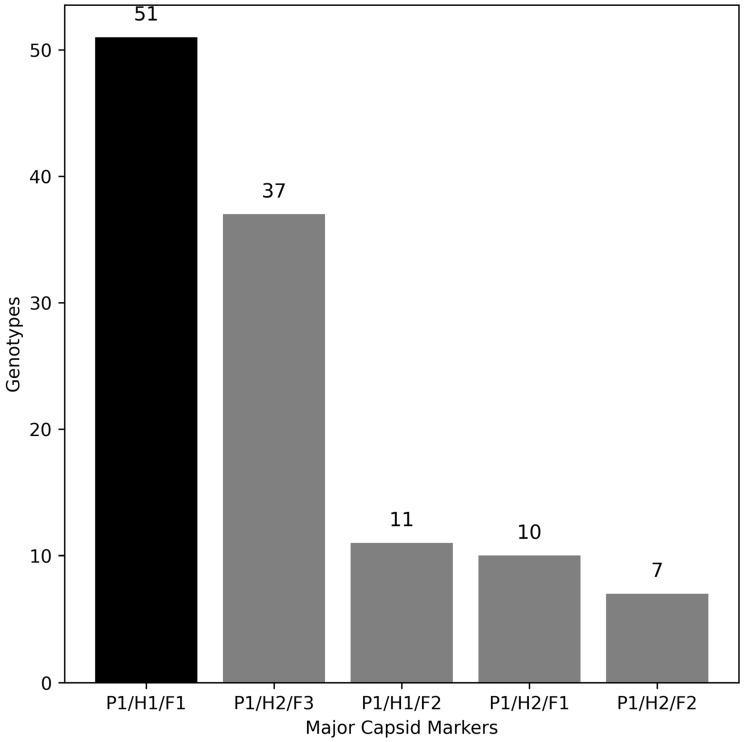
Recombinant HAdV genomes parsed by the three major capsid genes. Nonrecombinants are noted as P1/H1/F1 (black bar), representing the penton base, hexon, and fiber genes permutation unique from earlier prototypes. Recombinant genomes (gray bars) include P1/H2/F3, which are genomes that are derived from at least three parental genomes (that is, one parent was a recombinant); genomes with at least two parental contributors are designated as P1/H1/F2, P1/H2/F1, and P1/H2/F2. Genome sequences intergenic to the three major capsid genes are not scored under the current scheme.

A tally of the genotypes noted in Table 1 and Table 2 is presented in Figure 6. To date, there are 51 genomes that contain penton base, hexon, and fiber genes first described uniquely for an HAdV, noted on the histogram as P1/H1/F1; these are prototypes for reference. Sixty-five genomes are recombinant, with at least two parental genomes represented at the three major capsid genes. Thirty-seven are recombinants with at least three parentals, having unique combinations at the three major capsid genes, and are noted as P1/H2/F3. These may have a parental contributor that was a recombinant. Twenty-eight are recombinants with at least two parentals, noted as P1/H1/F2, P1/H2/F1, and P1/H2/F2. These are also prototypes for reference.

## 4. Discussion

Microbial, including viral, species and subspecies classification is important for identifying, characterizing, and understanding unknown pathogens in epidemics that occur, particularly pandemic outbreaks. The critical question of whether the emergent pathogen is truly novel or is a re-emergent and variant of a known pathogen may be answered through its classification, specifically via genome sequence. This, in turn, may provide clues for managing, limiting, and possibly preventing these outbreaks, as well as for more insightful understanding of the epidemiology of these pathogens. An example of applying Next-Generation Sequencing to identify and characterize pathogens responsible has been reported in the recognition of multispecies cocirculation of HAdVs during an acute gastroenteritis outbreak in 2023 [54].

Recently, a “Viral Subspecies Classification Workshop” was co-sponsored by the Bacterial and Virus-Bioinformatics Resource Center (BV-BRC), National Institute of Allergy and Infectious Diseases (NIAID)/National Institutes of Health (NIH), Centers for Disease Control and Prevention (CDC), and National Center for Biotechnology Information (NCBI), bringing together researchers across several virus families to discuss viral species and subspecies classification (NIAID facility; Rockville, MD, USA; 8–10 April 2024). As noted in several presentations, the availability and accessibility of public resources, including specific pathogen databases and websites, are key for up-to-date reference, identification, communications, and research. An example is presented in this report as a human adenovirus database: “Human Adenovirus Working Group Reference Website”. Others include the HBdb: The Hepatitis B Virus database (https://hbvdb.lyon.inserm.fr/HBVdb/HBVdbIndex, accessed on 1 February 2026) [55]; International Human Papillomavirus Reference Center (https://www.hpvcenter.se/, accessed on 1 February 2026) [56,57]; PaVE: The papillomavirus episteme (pave.niaid.nih.gov) [58]; and hepatitis C virus (HCV) database (https://hcv.lanl.gov/content/index, accessed on 1 February 2026) [59,60].

In addition to providing archival information, a reference and vetting working group site prevents conflicts of different novel isolates named simultaneously with the same genotype number. An example of this, prior to the formation of this resource, is the report of a novel human epidemic keratoconjunctivitis pathogen HAdV-D53 [22]. Upon publication, another “HAdV-D53” was “posted on-line” as an ePublication (May 2009) of a novel and different human epidemic keratoconjunctivitis pathogen. The latter was formally retracted as HAdV-D53 and republished as HAdV-D54 [61].

Another benefit of these public resources, along with using the genome as a basis for characterization, is to have a standardized naming system [20,21]. Prior to the acceptance of “genotype numbers” to represent different HAdV types, individual research-group-initiated, ad hoc names were published. For example, HAdV-D54 was originally published as “Kobe-H” in the literature and GenBank (AB333801) in June 2008 [62]. Another example is HAdV-58 [63], a novel gastrointestinal pathogen isolated in February 1996. Its designation was “Ad-Cor-96-487” when published originally in November 2009 [64]. Both are recognized as unique genotypes based on genome sequences and noted in the context of their penton base, hexon, and fiber genes, as shown in Table 2.

As a recent example of “nomenclature cacophony”, the 2022–2023 four-month very large HAdV outbreak in Kolkata (India) has been reported as involving multiple HAdV variants, including recombinants. It is not clear how many variants were identified and characterized, nor was it presented clearly if the more pathogenic variants were separate from the less pathogenic variants. Further, are these putative recombinants novel emergent genotypes? Are they variants of currently recognized circulating genotypes? The report states uninformatively “[isolates] belonged to type 3, displaying genetic constituent H7F3P7 and H3F3P7”; “type 7, of which 20 samples exhibited H7F3P7 genetic constituent and one sample showed H3F3P7 genetic constituent”; and “All the deceased cases were analyzed as adenovirus type 7 with H7F3P7 genetic constitution except one, which belonged to H3F3P7” [9]. As discussed earlier, a resolution to the multiple variants reported may be a recall of a previous identified, but without a “standard” name, respiratory pathogen from 1958 and 1995/2002 with serotype identity as both type 3 and type 7, isolated, characterized, and published several times as the “Takeuchi” or “AV3-7” or “AV3/7” strain in Japan [41,43]. Again, subsequent analysis with restriction enzyme digest ladders and serotyping indicate it as a recombinant with an epsilon epitope presenting as both HAdV-B3 and -B7 and a gamma epitope as HAdV-B3 [42].

The current format for designating the characterizing HAdV genome markers is penton base, hexon, and fiber (P/H/F) to reflect their genomic locations. For global public health surveillance and communications to the research community in the literature, adherence to currently accepted and standardized nomenclature (and pathogen characterization) is a must! In the case of the Takeuchi strain, a new hexon type number would be appropriate, as well as a novel genotype number.

In response to a “*frequently asked question*” over the years, it is noted that basing genotype recognition solely on three markers is not ideal, as other genomic sequences and regions are also important in the context of HAdV biology, e.g., HAdV replicative protein NFI binding site [21,22,23], host–pathogen interactions, e.g., the E3 genes [26,27,65,66,67], and viral pathogenicity, e.g., E1B-55K gene [68]. However, at the time in 2010, it was the scheme “*acceptable*” to the serologists, although this apparently remains still “controversial” [19,20,21]. Also noted is the absence of a method to distinguish novel genotypes based on serological differences in the epsilon and gamma epitopes, as well as the penton base variable sequences [53,69,70]. Again, this has been difficult to establish and to automate, as noted in Section 2.

With respect to nomenclature and a much broader and high-resolution overview and perspective, coupled with the release of whole genome data from members of the nonhuman simian species, that is, both captive [71,72,73,74,75,76,77] and noncaptive [30,77,78,79,80,81,82], strong support for zoonosis as a mechanism in the emergence of novel human adenoviral pathogens is evident, as noted in reports for two human HAdV pathogens arising through host species crossovers [28,29,30]. Likely this is a two- and multi-way route [28,29] that includes anthropozoonosis and zooanthroponosis [28,29,30,75,77,83]. In contrast to the thoughts of the members of the International Committee on Taxonomy of Viruses (ICTV) working group on adenoviruses, there is one single phylogenetic tree for both human and nonhuman simian adenoviruses as proposed recently [49], with designations for specific hosts such as “GoAdV” for gorilla adenoviruses and “ChAdV” for chimp adenoviruses for clarity, rather than the generic “SAdV” for all nonhuman simian adenoviruses.

The importance and utility of public resources such as this “Human Adenovirus Working Group Reference Website” (http://hadvwg.gmu.edu/, accessed on 1 February 2026) is providing an example of how a readily available overview and perspective of human adenovirus genotypes can provide insights into the emergence of novel human double-stranded DNA viral pathogens. Prior to and concurrent with adenoviral nucleic acid sequencing [84,85], there were debates of whether HAdV genomes recombined. Even into the 1990s, this was controversial, as one manuscript reporting HAdV coinfections required additional experimental data prior to publication [86]. As noted in Table 1 and Table 2, the prototypes originally designated as serotypes (HAdV-C1 through D51) have been whole-genome sequenced, analyzed, and compiled along with recent newly identified (non-serotyped) isolates (HAdV-G52 through D116). Of the 51 HAdV-C1 through D51 prototypes, genomic analysis revealed several are recombinants, with important changes in pathogenicity. HAdV-B16 contains a hexon derived from HAdV-E4 [25,87], an “exception proving the rule” that adenoviral recombination does not occur between human adenoviral species. HAdV-D15 and HAdV-D29 contain highly similar hexons and different fibers [88]. Genomic analysis of an ATCC sample of HAdV-D30 revealed two viruses, with highly similar hexons; one was renamed HAdV-D63 [52]. In the context of these two or three exceptions, 49 prototypes provide the reference sequences for the penton base, hexon, and fiber genes.

In contrast, Table 2 presents emergent and novel HAdVs, beginning with HAdV-G52, a gastrointestinal pathogen [46]. In this collection of “recently” isolated and characterized HAdV, it contains a completely “novel” genome that is nonrecombinant. Although it has not been observed subsequently in either human or nonhuman simian samplings, a report suggesting it is a zoonotic pathogen also indicates it is either a donor or a recipient in a recombination event involving a rhesus macaque adenovirus [30].

On the other hand, from HAdV-D53 to -D116, 63 genotypes are characterized as recombinants, containing one or two of the “landmark” genes from previously reported genotypes. The single nonrecombinant is HAdV-D62. These emergent, novel human pathogens presented with host–virus differences from the parental prototypes with respect to pathogenicity attributes. For example, HAdV-D53 is a then-emergent ocular pathogen, in which one parental epitope derived from and provided hexon identification as HAdV-D22; HAdV-D22 is not a pathogen [22]. This is similar to the identification of another ocular pathogen, HAdV-D64, originally serotyped as HAdV-D19, which is nonpathogenic [89]. Therefore, with respect to genome mutations, recombination may result in emergent and significant human pathogens, at times unnoticed due to serological mischaracterization. As an example of this is a report in 2011 in which the recently reported recombinant HAdV-D53, which was isolated in Germany [22], was reported in several outbreaks in Japan [90]. Furthermore, the authors noted HAdV-D53 “has recently become the third most commonly detected strain in EKC patients in Japan….apparently misidentified as HAdV-D8, -D22, or -D37” [90]. To illustrate this was not a unique instance, a 2011 report by H. Kaneko, et al. of the analysis and reanalysis of archived EKC pathogens in Japan and “six other countries”, another recently identified recombinant HAdV-D54, previously mistyped using serology as HAdV-D8, was noted as “commonly been detected in samples from epidemic keratoconjunctivitis (EKC) patients”. To underscore further the importance of recombination in the emergence of novel human pathogens, “HAdV-54 has been isolated each year since 1995” [61].

Another striking example is HAdV-B55, which was serotyped as HAdV-B11, a renal pathogen [20,91]. In contrast, HAdV-B55 is a highly contagious respiratory tract pathogen linked to several large outbreaks and associated with high morbidity rates, particularly in China [7]. The recombination event resulting in a presumably less commonly circulating hexon epitope type presenting to an immune-naïve population is akin to a Trojan horse. HAdV-B66 [48,92] was originally characterized serologicaly as HAdV-B3 based on its hexon epitope, then retyped as HAdV-B7; genomic analysis revealed it as a recombinant, with parental HAdV-B3 and -B7. Significantly, it was associated with several outbreaks linked to high mortality rates in South America [93].

Finally, an unexpected and intriguing observation of a “promiscuous” hexon gene is noted. In several novel, emergent HAdV ocular pathogens, HAdV-D56, -D69, -D82, -D87, -D88, and -D94, their hexon genes are recombined from a parental or ancestral HAdV-D15, in addition to HAdV-D29 [88]. The recombination mechanism may be due to shared proteins or sequences favoring this exchange [94]. A rationale for the selection of this particular hexon is yet to be determined.

As presented in Figure 6, the number of recombinants (65) outnumber the number of nonrecombinant HAdVs (51) currently. Beyond the initial set of 51 HAdV prototypes, only two are nonrecombinants (HAdV-G52 and -D62). These recombinants have been reported in subsequent patients and outbreaks, including archived and retyped isolates [90], with one, HAdV-B55, associated with several large outbreaks and higher morbidity and mortality [7]. Additionally, many recently reported recombinants are not associated with novel genotype numbers and not included in this tally, for example, the large outbreak in West Bengal, India [9]. Finally, recent reports include previously reported recombinants as well as novel recombinants to be submitted to the working group (N.D. Yolshin, *pers. communications)* [95].

There is a breadth and depth in the multitude of genome data from variants observed across the individual genotypes, including spatial and temporal (archival) isolates. HAdVs are large double-stranded DNA-based viruses that are encoded by genomes that are generally stable with respect to base substitutions and deletions and insertions (indels). For example, the HAdV-C5 prototype genome has been sequenced from a low-passaged laboratory reference strain (“Adenovirus Reference Material” (“ARM”)), as well as from then-current circulating laboratory and “field” isolates [96,97]. The evidence from HAdV-C5 shows that the double-stranded DNA human adenoviral genomes are relatively stable over many replicative cycles across 45 years [96] and are reflective of the fidelity of the DNA polymerase replication machinery, in contrast to that of the genomes of the RNA-based viruses and their replicative polymerases [98]. A recent report using a genomics approach supports this observation, revealing that the HAdV-C5 low mutation rate results from a combination of the intrinsic replication fidelity of its DNA polymerase and the host cell post-replicative repair mechanisms, and that it was “similar to other large double-stranded DNA viruses” [99]. Complementing this is a recent review of the population genetics of virus mutation rates across the spectrum of types of genomes [100]. The overview of emergent HAdV genotypes, visualized through the tables and figures presented in this report is facilitated by the availability of the “Human Adenovirus Working Group” (http://hadvwg.gmu.edu/, accessed on 1 February 2026) web-based resource. These provide an interesting perspective to the molecular evolution of this virus, that is, the emergence of novel genotypes and human pathogens appears driven mainly by genome recombination or, imaginatively, exchanging genomic space via “castling”. Considering that these recent novel genotypes and emergent pathogens emerged via this approach and that this appears diametrically opposed to the evolution strategy of the “Red Queen Reigns in the Kingdom of RNA Viruses” exemplified in the 1990s by vesicular stomatitis virus [31], we propose an evolution strategy for this double-stranded DNA virus that results in an equally and dynamically changing genome as the “White King Reigns in the Family of Human Adenoviruses”.

## Figures and Tables

**Table 1:** Summary of human adenovirus genotypes HAdV-C1 through -G52, formerly termed serotypes. Note, HAdV-G52 was the first HAdV recognized and proposed as a novel genotype based on whole genome data rather than serological data. In the table, the genotype nomenclature includes a species designation A–G. The column “Name” provides designations that incorporate the genes for the three major capsid proteins (P for penton base; H for hexon; and F for fiber), whose genes span the genome and provide convenient markers for visualizing genome recombinants. Accession numbers are GenBank reference, archival, and retrieval designations. Genome data publications are noted by the “Year of Publication”. Penton base, hexon, and fiber type numbers, referencing the first prototype from which they were identified, are noted in the final three columns.

Adenovirus Genotype	Name	Accession No.	Year (Publication)	Penton Base	Hexon	Fiber
HAdV-C1	P1H1F1	AF534906	2004	1	1	1
HAdV-C2	P2H2F2	JX173081.1	2002	2	2	2
HAdV-B3	P3H3F3	AY599834	2004	3	3	3
HAdV-E4	P4H4F4	AY594253	2005	4	4	4
HAdV-E4.2	P4H4F4	AY599835	2006	4	4	4
HAdV-C5	P5H5F5	AY339865	2003	5	5	5
HAdV-C6	P6H6F6	FJ349096	2008	6	6	6
HAdV-B7	P7H7F7	AY594255	2005	7	7	7
HAdV-D8	P8H8F8	LC312462.1	2009	8	8	8
HAdV-D9	P9H9F9	AJ854486	2004	9	9	9
HAdV-D10	P10H10F10	JN226746	2011	10	10	10
HAdV-B11	P11H11F11	AY163756	2003	11	11	11
HAdV-A12	P12H12F12	X73487.1	1994	12	12	12
HAdV-D13	P13H13F13	JN226747	2011	13	13	13
HAdV-B14	P14H14F14	AY803294	2004	14	14	14
HAdV-D15	P15H15F15	AB562586	2010	15	15	15
HAdV-B16	P16H4F16	AY601636	2004	16	4	16
HAdV-D17	P17H17F17	HQ910407	2011	17	17	17
HAdV-A18	P18H18F18	GU191019	2010	18	18	18
HAdV-D19	P19H19F19	JQ326209	2011	19	19	19
HAdV-D20	P20H20F20	JN226749	2013	20	20	20
HAdV-B21	P21H21F21	AY601633	2006	21	21	21
HAdV-D22	P22H22F22	FJ619037	2009	22	22	22
HAdV-D23	P23H23F23	JN226750	2013	23	23	23
HAdV-D24	P24H24F24	JN226751	2013	24	24	24
HAdV-D25	P25H25F25	JN226752	2013	25	25	25
HAdV-D26	P26H26F26	EF153474	2007	26	26	26
HAdV-D27	P27H27F27	JN226753	2013	27	27	27
HAdV-D28	P28H28F28	FJ824826	2010	28	28	28
HAdV-D29	P29H29F29	JN226754	2013	29	15	29
HAdV-D30	P30H30F30	JN226755	2012	30	30	30
HAdV-A31	P31H31F31	AM749299	2005	31	31	31
HAdV-D32	P32H32F32	JN226756	2013	32	32	32
HAdV-D33	P33H33F33	JN226758	2013	33	33	33
HAdV-B34	P34H34F34	AY737797	2004	34	34	34
HAdV-B35	P35H35F35	AY271307.1	2003	35	35	35
HAdV-D36	P36H36F36	GQ384080	2010	36	36	36
HAdV-D37	P37H37F37	DQ900900.1	2009	37	37	37
HAdV-D38	P38H38F38	JN226759	2013	38	38	38
HAdV-D39	P39H39F39	JN226760	2011	39	39	39
HAdV-F40	P40H40F40	KU162869.1	1993	40	40	40
HAdV-F41	P41H41F41	DQ315364	2007	41	41	41
HAdV-D42	P42H42F42	JN226761	2013	42	42	42
HAdV-D43	P43H43F43	JN226762	2013	43	43	43
HAdV-D44	P44H44F44	JN226763	2013	44	44	44
HAdV-D45	P45H45F45	JN226764	2011	45	45	45
HAdV-D46	P46H46F46	AY875648	2004	46	46	46
HAdV-D47	P47H47F47	JN226757	2011	47	47	47
HAdV-D48	P48H48F48	EF153473	2006	48	48	48
HAdV-D49	P49H49F49	DQ393829	2006	49	49	49
HAdV-B50	P50H50F50	AY737798	2004	50	50	50
HAdV-D51	P51H51F51	JN226765	2011	51	51	51
HAdV-G52	P52H52F52	DQ923122	2005	52	52	52

**Table 2:** Summary of human adenovirus genotypes HAdV-D53 through -D116, as prototypes based on their genome sequences. The table nomenclature includes a species designation A–G. In the table, the genotype nomenclature includes a species designation A–G. The column “Name” provides designations that incorporate the three major capsid proteins (P for penton base; H for hexon; and F for fiber), whose genes span the genome and provide convenient markers for visualizing genome recombinants. Accession numbers are GenBank reference, archival, and retrieval designations. Note: Although accession numbers for HAdV-C89, -D90, and -D116 have been assigned, the researchers have chosen to withhold details until the formal publication of their findings. Genome data publications are noted by the “Year of Publication”. Penton base, hexon, and fiber type numbers, referencing the first prototype from which they were identified, are noted in the final three columns. * HAdV-D15 hexon is noted for several emergent, novel genotypes.

Adenovirus Genotype	Name	Accession No.	Year (Publication)	Penton Base	Hexon	Fiber
HAdV-D53	P37H22F8	FJ169625	2009	37	22	8
HAdV-D54	P54H54F8	AB333801	2009	54	54	8
HAdV-B55	P14H11F14	FJ643676	2009	14	11	14
HAdV-D56	P56H15F9	HM770721	2010	56	15 *	9
HAdV-C57	P1H57F6	HQ003817	2011	1	57	6
HAdV-D58	P58H58F29	HQ883276	2011	58	58	29
HAdV-D59	P64H25F56	JF799911	2012	64	25	56
HAdV-D60	P60H20F60	HQ007053	2013	60	20	60
HAdV-A61	P31H61F31	JF964962	2011	31	61	31
HAdV-D62	P62H62F62	JN162671	2014	62	62	62
HAdV-D63	P30H30F29	JN935766	2012	30	30	29
HAdV-D64	P22H19F37	EF121005	2006	22	19	37
HAdV-D65	P58H10F9	AP012285	2012	58	10	9
HAdV-B66	P7H7F3	JN860676	2012	7	7	3
HAdV-D67	P67H9F67	AP012302	2013	67	9	67
HAdV-B68	P16H3F16	JN860678	2011	16	3	16
HAdV-D69	P37H15F69	JN226748	2013	37	15 *	69
HAdV-D70	P70H70F29	KP641339	2015	70	70	29
HAdV-D71	P9H20F71	KF268207	2013	9	20	71
HAdV-D72	P72H30F72	KF268335	2013	72	30	72
HAdV-D73	P67H45F27	KY618676	2017	67	45	27
HAdV-D74	P70H74F51	KY618677	2017	70	74	51
HAdV-D75	P75H26F29	KY618678	2017	75	26	29
HAdV-B76	P21H21F16	KF633445	2013	21	21	16
HAdV-B77	P35H34F7	KF268328	2013	35	34	7
HAdV-B78	P11H11F7	KT970441	2016	11	11	7
HAdV-B79	P11H34F11	LC177352	2016	11	34	11
HAdV-D80	P19,23H28F22	KY618679	2022	22	28	22
HAdV-D81	P65H48F60	AB765926.1	2014	65	48	60
HAdV-D82	P56H15F37	LC066535.1	2015	56	15 *	37
HAdV-D83	P83H9F15	KX827426.1	2016	83	9	15
HAdV-D84	P43H17F84	MF416150	2017	43	17	84
HAdV-D85	P37H19F8	LC314153	2018	37	19	8
HAdV-D86	P9H25F25	KX868297	2018	9	25	25
HAdV-D87	P9H15F25	MF476841	TBA	9	15 *	25
HAdV-D88	P88H15F9	MF476842	TBA	88	15 *	9
HAdV-C89	P89H2F2	TBA	2019	89	2	2
HAdV-D90	P33H27F67	TBA	TBA	33	27	67
HAdV-D91	P37H37F17	KF268208	2019	37	37	17
HAdV-D92	P92H32F27	KF268325	2019	92	32	27
HAdV-D93	P28H37F38	KF268334	2019	28	37	38
HAdV-D94	P33H15F9	KF268201	2019	33	15 *	9
HAdV-D95	P95H9F15	KF268206	2019	95	9	15
HAdV-D96	P23H32F62	KF268327	2019	23	32	62
HAdV-D97	P67H28F60	KF268320	2019	67	28	60
HAdV-D98	P98H46F9	KF268332	2019	98	46	9
HAdV-D99	P9H46F39	KF268211	2019	9	46	39
HAdV-D100	P100H17F30	KF268330	2019	100	17	30
HAdV-D101	P101H37F45	KF268324	2019	101	37	45
HAdV-D102	P102H38F30	KF268312	2019	102	38	30
HAdV-D103	P103H33F30	KF268322	2019	103	33	30
HAdV-C104	P1H1F2	MH558113	2021	1	1	2
HAdV-D105	P105H59F9	ON393913	2020	105	59	9
HAdV-B106	P11H11F35	ON393912	2020	11	11	35
HAdV-D107	P102H107F25	MK174992	2010	102	107	25
HAdV-C108	P1H2F2	ON054624	2014	1	2	2
HAdV-D109	P22H19F9	OM830314	2018	22	19	9
HAdV-D110	P67H110F9	OM830315	2018	67	110	9
HAdV-D111	P37H9F9	LC652931	2016	37	9	9
HAdV-D112	P112H112F67	OQ679041	2018	112	112	67
HAdV-D113	P20H42F42	MW694832	2021	20	42	42
HAdV-B114	P7H3F3	OR853835	2023	7	3	3
HAdV-D115	P22H8F8	OR044915	2024	22	8	8
HAdV-D116	P33H28F71	TBA	2024	33	28	71

## Data Availability

Data for recently recognized novel HAdV genotypes are available at http://hadvwg.gmu.edu/ (accessed on 1 February 2026), with the genome data archived at GenBank (https://www.ncbi.nlm.nih.gov/genbank/, accessed on 1 February 2026).

## Note Added in Proof

To underscore the dynamic nature of HAdV evolution and the role of recombination in driving the emergence of new types, HAdV-B117 (P7/H3/F7) has been added to the recognized genotypes; an additional candidate, HAdV-D118 (P20/H20/F42), is currently under review.
